# Pattern of Mutation Rates in the Germline of *Drosophila melanogaster* Males from a Large-Scale Mutation Screening Experiment

**DOI:** 10.1534/g3.114.011056

**Published:** 2014-06-11

**Authors:** Jian-Jun Gao, Xue-Rong Pan, Jing Hu, Li Ma, Jian-Min Wu, Ye-Lin Shao, Shi-Meng Ai, Shu-Qun Liu, Sara A. Barton, Ronny C. Woodruff, Ya-Ping Zhang, Yun-Xin Fu

**Affiliations:** * Laboratory for Conservation and Utilization of Bio-Resources, Yunnan University, Kunming, Yunnan, China; † Division of Biostatistics and Human Genetics Center, School of Public Health, The University of Texas at Houston, Houston, Texas; ‡ Department of Biological Sciences, Bowling Green State University, Bowling Green, Ohio; § State Key Laboratory of Genetic Resources and Evolution, Kunming Institute of Zoology, Chinese Academy of Sciences, Kunming, Yunnan, China

**Keywords:** *Drosophila melanogaster*, cell coalescent, germline mutation rate, likelihood inference

## Abstract

The sperm or eggs of sexual organisms go through a series of cell divisions from the fertilized egg; mutations can occur at each division. Mutations in the lineage of cells leading to the sperm or eggs are of particular importance because many such mutations may be shared by somatic tissues and also may be inherited, thus having a lasting consequence. For decades, little has been known about the pattern of the mutation rates along the germline development. Recently it was shown from a small portion of data that resulted from a large-scale mutation screening experiment that the rates of recessive lethal or nearly lethal mutations differ dramatically during the germline development of *Drosophila melanogaster* males. In this paper the full data set from the experiment and its analysis are reported by taking advantage of a recent methodologic advance. By analyzing the mutation patterns with different levels of recessive lethality, earlier published conclusions based on partial data are found to remain valid. Furthermore, it is found that for most nearly lethal mutations, the mutation rate at the first cell division is even greater than previous thought compared with those at other divisions. There is also some evidence that the mutation rate at the second division decreases rapidly but is still appreciably greater than those for the rest of the cleavage stage. The mutation rate at spermatogenesis is greater than late cleavage and stem-cell stages, but there is no evidence that rates are different among the five cell divisions of the spermatogenesis. We also found that a modestly biased sampling, leading to slightly more primordial germ cells after the eighth division than those reported in the literature, provides the best fit to the data. These findings provide conceptual and numerical basis for exploring the consequences of differential mutation rates during individual development.

Sperm from any organism results from a series of cell divisions during individual development, and each of these divisions may lead to mutations (Gilbert 2003). Because mutation is the ultimate cause of genetic variation, numerous studies have focused on understanding various aspects of mutation, including estimations of its rate. The overall mutation rate per generation is an essential quantity for various evolutionary/population genetic studies and is thus the focus of much research. However, a detailed understanding of mutation rates along individual development is also indispensable and an integral knowledge of biology and its importance has been widely recognized in medical genetics, particularly in the study of cancer/tumor development. A mutation that occurred early in the development will likely lead to more descendants (somatic or germ cells) than one that occurred later and thus will likely have more impact on the host as well as on its chance of survival in the population (Woodruff *et al.* 1996; Huai and Woodruff 1997; Woodruff and Thompson 2005). Differential mutation rates during development also provide new insights on male-driven evolution (Gao *et al.* 2011).

Until recently, there has been little progress on dissecting mutation rates during germline development at the level of individual cell divisions. Although next-generation sequencing may hold great promise for providing rich information for such purposes, well-developed classic experiments can still be a powerful and cost-effective approach, particularly for some model organisms. Gao *et al.* (2011) reported an analysis of the mutation patterns from a large-scale experiment for screening recessive lethal or nearly lethal mutations and found that mutation rates differ substantially in the germline lineage. In particular, the first cell division harbors the greatest mutation rate, followed by the divisions in spermatogenesis, whereas cell divisions in between have at least a magnitude smaller mutation rate. Gao *et al.* (2011) analyzed only those mutations of extreme recessive lethality, which are only a small fraction of the available data, due to a technical difficulty that also limited the analysis to families of exactly 20 offspring with at most two mutations per family. Fu (2013) extended the previous inference framework (Gao *et al.* 2011) with a new method for approximating the probability of a mutation pattern and a refined coalescent algorithm for simulating sample genealogies which are necessary for deriving coefficients used in the analysis. The refined inference framework not only removes the limitation of at most two mutations per families but also allows families of different sizes. This framework also established the confidence for conclusions derived from likelihood ratios based statistical tests for mutation screening data.

Taking advantage of the aforementioned methodologic progress, we report in this paper the analysis of the complete data set from the experiment, which consists of 9872 families of various sizes and which contains three times more mutations than previously reported. The greater resolution of the data as well as the contrast of results from analyzing mutations with different recessive lethalities allows us to obtain more accurate/stable estimates of mutation rates, to explore some aspects of the mutation process, and to test hypotheses that were unattainable previously. As a result, a deeper understanding of the mutation rate patterns along germline development is achieved. Furthermore, new hypotheses are presented and the male-driven hypothesis is re-evaluated in light of the new results from the current analysis.

## Materials and Methods

### Materials

*Drosophila melanogaster* stocks from Woodruff’s laboratory in Bowling Green State University were used. These flies were maintained by taking advantage of the balancer chromosomes that were pioneered by H. J. Muller (Muller 1928) for the purpose of maintaining newly isolated mutations, including recessive lethals, without selection (Muller and Oster 1963; Abrahamson and Lewis 1971; Ashburner 1989; Greenspan 1997). Balancers for each of the major chromosomes of these *D. melanogaster* contain multiple inversions and one or more dominant visible mutations. The inversions, which were mapped by using giant polytene chromosomes, act as crossover suppressors and the clearly visible dominant mutations allows for the identification of heterozygotes. With these chromosome stocks, new lethal or nearly lethal mutations are maintained in the heterozygous state against the balancer chromosomes without the concern of being lost due to recombination. The experiment employed three types of autosomal haploid chromosomes (genomes), which are denoted by *β*, *γ*, and z. More specifically they are as follows:

$$\begin{array}{l} \beta =\text{T}\left(2;3\right)\text{A}1-\text{W},\;\mathrm{Cy}\;L\;\mathrm{Ubx}\\ \gamma =\text{T}\left(2;3\right)\text{B}18,\;\mathrm{Pm}\;\mathrm{Sb}\\ z=+;+. \end{array}$$

The *β* type balancer is homozygous lethal and is marked with the dominant visible, and recessive lethal mutations, Curly (*Cy*) wings, Lobe (*L*) eye, and Ultrabithorax (*Ubx*) enlarged halteres. This balancer segregates as a unit and suppresses crossing over on both the second and third chromosomes effectively (Lindsley and Zimm 1992; Thompson 1977). Similarly, the *γ* chromosome is also homozygous lethal and carries dominant visible markers. Type z represents a haploid genome with wild-type second and third chromosomes that are free of lethal mutations at the start of experiment. Recessive lethal or nearly lethal mutations in z are the screening target of the experiment.

### Experiment

Graf *et al.*’s (1992) methods for culturing the flies were followed with some modifications. Drosophila medium containing water, glucose, agar, corn meal, and the antifungal agent methyl-p-hydroxybenzoate was cooked and dispensed into glass culture vials (10 cm in height and 3.5 cm in diameter) via a self-made dispenser. Also, self-made vial holders (designed to hold vials in a 10 × 10 array to match the dispenser) were used to facilitate the work. After drying and cooling the medium, a small piece of sterilized filter paper was folded and inserted into the medium with forceps to increase the surface and to regulate humidity within the vial, and then a small amount of live baker’s yeast was seeded into the vial. The culture vials, with cotton plugs added, were used to start the cultures. The flies were reared in a chamber, which allowed simultaneous culturing of more than 20,000 culture vials of flies under standard conditions (25°, approximately 60% relative humidity and 16-hr light:8-hr dark). The temperature in the culturing chamber was adjusted with air-conditioners and a self-regulating electric heating system, and humidity was manually controlled with a humidifier.

As briefly described in Gao *et al.* (2011), the mutation screening experiment, which is similar to protocols that have been used in various laboratories (Thompson and Woodruff 1980; Woodruff *et al.* 1984; Mason *et al.* 1985; Brodberg *et al.* 1987; Woodruff *et al.* 1996), consists of two parts. The first is to employ a three-generation assay to identify autosomal-recessive lethal or nearly lethal mutations in approximately 1200 genes on the second and third chromosomes in *D. melanogaster*. The second component of the experiment is known as the *allelism test*, which is to delineate the identity of mutations leading to different mutants.

The first component of the experiment was designed to screen *β*/z male offspring of crosses between single *β*/z males and multiple *β*/*γ* virgin females to see whether a new lethal or nearly lethal mutation occurred in the z chromosomes during germline development of the father. In essence, the mating scheme within each family derived from a single *β*/z male is as follows:

$$\begin{array}{l} \mathrm{Parental}:\text{Multiple}\;\beta /\gamma \;\text{virgin}\;♀\times \text{single}\;\beta /\text{z}\;♂\\ \;\;\;\;\left(20-35\;\beta /\text{z}\;♂\;\text{offspring}\;\text{were}\;\text{each}\;\text{subjected}\;\text{to}\;\text{the}\;\text{following}\;\text{assay}\right)\\ \;\;\;\;\;F_{1}:\text{Multiple}\;\beta /\gamma \;\text{virgin}\;♀\times \text{single}\;\beta /\text{z}\;♂\\ \;\;\;\;\left(\text{multiple}\;\beta /\text{z}\;♀\;\text{and}\;♂\;\text{were}\;\text{obtained}\;\text{and}\;\text{were}\;\text{used}\;\text{for}\;\text{the}\;F_{2}\right)\\ \;\;\;\;\;F_{2}:\text{Multiple}\;\beta /\text{z}\;\text{virgin}\;♀\times \text{multiple}\;\beta /\text{z}\;♂\\ \;\;\;\;\;F_{3}:\text{Identify}\;\text{the}\;\text{genotype}\;\text{of}\;\text{each}\;\text{surviving}\;\text{offspring},\;\text{which}\;\text{is}\;\text{either}\;\beta /\text{z}\;\text{or}\;\text{z}/\text{z}. \end{array}$$

The intention here was to follow 20 offspring (lines) per family, but because some lines would not succeed for a variety of reasons, including death and failing quality control, up to 35 lines were initiated per family. As a result, the number of offspring in a family ranged from 2 to 35. Parental flies were removed from culture vials well before we examined the progeny and collected virgin females. The progeny adult flies were etherized using the Drosophila Fly Anesthetizer (Burco) and examined for phenotype and sex under a stereo microscope. Virgin *β*/*γ* or *β*/z females were collected within about 8 hr after we removed parental flies and then collection was repeated every 8 hr (usually at about 8:00 am, 4:00 pm, and midnight) during the eclosion.

Care was taken to ensure that *β*/*γ* females were virgin during the experiment. As a quality control measure, if *β*/*γ* offspring in the *F*_3_ were observed, the line was deemed disqualified because such an event can only result from nonvirgin *β*/z females. Also, to make sure the degree *d* of recessive lethality or simply *lethality* (Fu 2013), which is one minus the percent of z/z homozygote among all offspring, is adequately estimated, 40 offspring were set to be the minimum number of offspring examined in *F*_3_. A line is declared to be tentatively a mutant with lethality *d* if the percentage of its z/z homozygotes among all surviving offspring is not larger than *d*.

The *β*/z males used in the Parental stage were selected from families of *F*_3_ in which no mutation of detectable lethality was found (*i.e.*, the percentage of z/z offspring is normal). In addition, the selection favored vigorous young males with distinct phenotypes. The process ensured that the chromosome z in the *β*/z male was devoid of recessive lethal mutation in general, but newly arisen recessive lethal mutation in F3 may escape such surveillance. The counting of *F*_3_ offspring was performed when there were a sufficient number of matured offspring. By the time a line was judged to be devoid of the targeted mutations, it would be typically 2−4 d after the minimum age for a matured adult.

The second component of the experiment is the *allelism test*, which is to delineate the identity of the mutation leading to each mutant. This was achieved by a series of crosses between mutant lines, with one line contributing *β*/z males and another virgin *β*/z females. There are usually many ways the crosses can be arranged, but each mutant line must be involved in at least one cross. The percentage of z/z individuals among offspring of a cross is expected to be similar to those of the parental lines if the mutations in the two different lines are the same and significantly higher when they are different (if mutations are indeed recessive). When there are multiple mutant lines in a family, a series of interconnected crosses between different mutant lines are necessary to resolve ambiguities.

### Statistical method

Using the same notation as Fu (2013), the information conveyed by mutation(s) in a family can be represented by a mutation pattern

κ=〈i,j,k…〉 (1)

in which each element represents a mutation and its value is the number of offspring carrying the mutation, or simply the *size of the mutation*. After a line obtains a mutation of lethality ≥ *d*%, a further mutation will likely do nothing or increase the level of lethality, but the effect is usually difficult to distinguish from the first one under our experimental setting. This often led to masking or nondetection of the second mutation (Fu 2013). As a result, each identified mutant offspring is associated with one and only one mutation in the mutation pattern. Therefore, there is a natural constraint that *i* + *j* + *k* … is not larger than the family size. The aggregation of mutation patterns for families of size *f* can be concisely represented as

$$f:\kappa_{1n\left(\kappa_{1}\right)}\kappa_{2n\left(\kappa_{2}\right)}\ldots .$$ (2)

where *n*(*κ_i_*) is the number of occurrences of pattern *κ_i_* and for brevity can be omitted if its value is 1. The statistical analysis of the experimental results consists of determining the mutation pattern in each family, estimating mutation rates, and testing hypotheses.

#### Determination of the mutation pattern in a family:

The log-likelihood ratio test is used to test the null hypothesis that two mutant lines share the same causal mutation against the alternative that they result from independent mutations. Under the null hypothesis, the likelihood is the product of three binomial distributions all having the same frequency for z/z offspring, while under the alternative the binomial distribution for the cross has different frequencies for z/z offspring. Suppose *z_i_* and *n_i_* are the numbers of z/z and total offspring for parental line *i*(*i* = 1, 2) respectively, and *z*_3_ and *n*_3_ are the corresponding numbers for the cross. Then the test statistic is

$$lr=-2\ln\left[\frac{p_{123}^{z_{1}+z_{2}+z_{3}}{\left(1-p_{123}\right)}^{n_{1}+n_{2}+n_{3}-z_{1}-z_{2}-z_{3}}}{p_{12}^{z_{1}+z_{2}}{\left(1-p_{12}\right)}^{n_{1}+n_{2}-z_{1}-z_{2}}\;p_{3}^{z_{3}}{\left(1-p_{3}\right)}^{n_{3}-z_{3}}}\right]$$ (3)

where *p*_123_ = (*z*_1_ + *z*_2_ + *z*_3_)/(*n*_1_ + *n*_2_ + *n*_3_), *p*_12_ = (*z*_1_ + *z*_2_)/(*n*_1_ + *n*_2_) and *p*_3_ = *z*_3_/*n*_3_. The test statistic follows asymptotically a χ^2^ distribution with one degree of freedom under the null hypothesis that the cross results in the same z/z percentage as the two parental lines. Therefore, a significant test result indicates that the two lines result from different mutations. Often, multiple crosses are performed among lines within a family. If the overall significance level is *α*, and there are *m* crosses, then the level of significance for each test should be set to about *α*/*m*. In general, larger values of *α* will result in more significant tests and thus more independent mutations will be inferred, but the number of mutants for a given lethality does not change significantly, which are mostly determined by the percentage of z/z offspring in the *F*_3_. The results presented in this paper corresponds to *α* = 0.10.

#### Estimating mutation rate and hypothesis testing:

**Figure 1:**
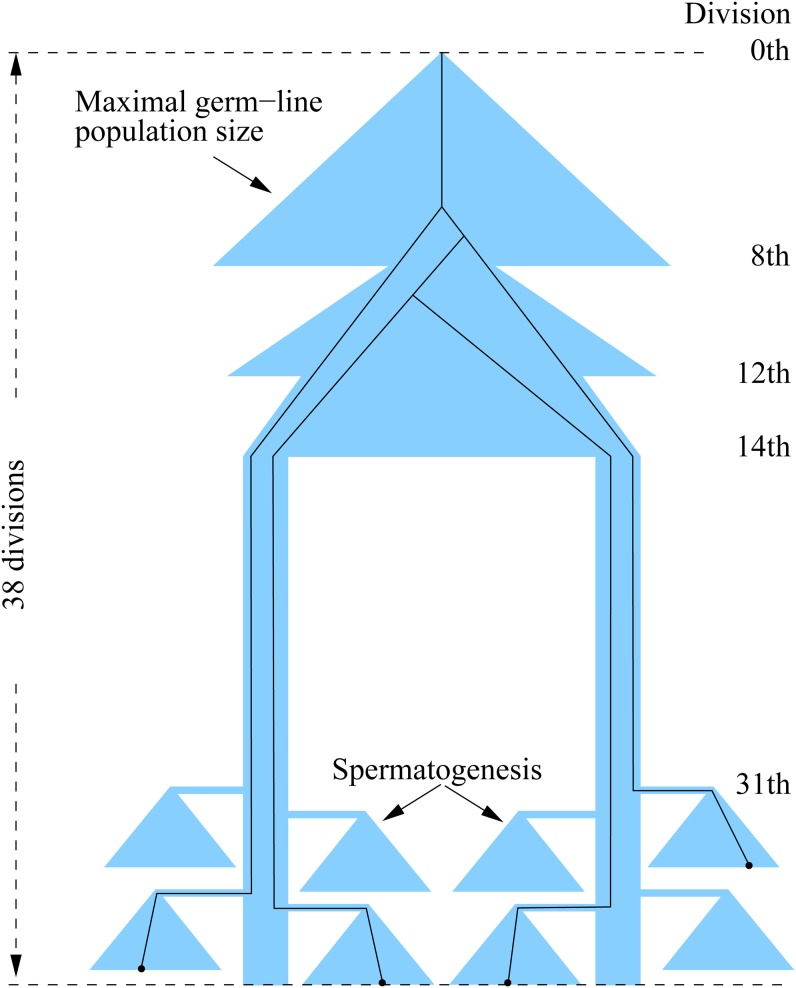
Population dynamics and an example of the genealogy of four sperm sampled at the time at which maximal 38th cell division has occurred (adapted from Fu 2013).

Suppose the development from the fertilized egg to the sperms are divided into *I* consecutive time intervals [*t_i_*_− 1_ + 1, *t_i_*] (i = 1,…I) (*t*_0_ = 0 and *t_I_* = maximum number of cell divisions), and let *u_i_* be the mutation rate per cell division within the ith interval. The estimate of ***μ*** = (*u*_1_, … *u_I_*) and statistical tests about them were performed through the maximum likelihood approach developed in Gao *et al.* (2011) and further refined in Fu (2013). The inference makes use of the information about population dynamics, intervals of cell divisions, and coalescent structure of the sample genealogy (Figure 1), but in essence, the inference framework is based on evaluating the likelihood function

$$L=\prod_{f}\prod_{\kappa }p_{f}{\left(\kappa \right)}^{n_{f}\left(\kappa \right)}$$ (4)

where *f* represents family size, *κ* is a mutation pattern for a given family size *f*, *n_f_*(*κ*) is the number of occurrences of pattern *κ* in families of size *f*, and *p_f_*(*κ*) is the probability of mutation pattern *κ*, which is a function of *μ* and coefficients that are derived from the dynamics of the germline population. The probability can be computed through a combined approach of both analytic derivation and coalescent simulation of samples. The first product enumerates over all the different family sizes, while for each family size the second product enumerates over all observed patterns. The maximum likelihood estimate $\hat{\mu }$ is the value of *μ*that maximizes the above likelihood function.

The overall mutation rate *μ* is defined as the sum of mutation rates over all divisions in the development. Thus the maximum likelihood estimate of *μ* is

$$\hat{\mu }=\sum_{i=1}^{I}\left(t_{i}-t_{i-1}\right){\hat{u}}_{i}.$$ (5)

Alternatively, an unbiased estimate of *μ* can be obtained by a classical approach (Fu and Huai 2003), which is

$$\tilde{\mu }=\frac{\#\;\text{of}\;\text{mutants}}{\#\;\text{of}\;\text{lines}}.$$ (6)

This estimate has the advantage of being unbiased regardless of the assumptions made on the dynamics of the germline lineage development.

## Results

### Data and summary

**Figure 2:**
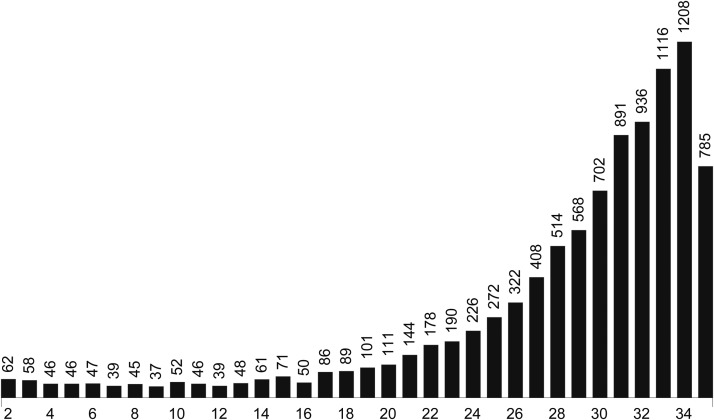
Distribution of family sizes in 9594 screened families.

More than 10,000 families were screened in a 4-yr period (2004−2008), from which 9872 succeeded with at least one survival line at *F*_3_. To obtain positional information about observed mutation(s), we further require at least two completed lines for a family. To make sure *β*/*γ* females in *F*_2_ are virgin, we further require that no *β*/*γ* offspring among the *F*_3_ was observed. Furthermore, when the recessive lethality of a line exceeds a given threshold and the identity of the mutation cannot be determined, the line was also removed. This occurs when such a line was not used in crosses with other mutant lines (if they exist). After the cleanup, 9594 families passed the quality control, which resulted in a total of 271,794 lines, which is 90% of the 300,737 examined lines. The mean number of lines per families is 28.3 and the mean number of offspring in the qualified lines in *F*_3_ is 117.9. The distribution of the family sizes is given in Figure 2.

**Figure 3:**
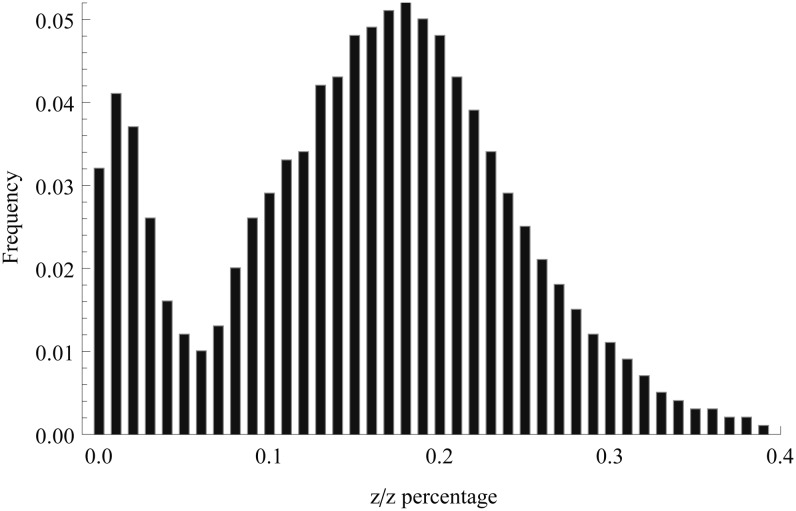
Distribution of the percentage of z/z offspring among 271,794 lines in *F*_3_.

The histogram of the percentage of z/z offspring in the 271,794 lines is given in Figure 3. There are two obvious modes in the histogram, one at 1% and another at 18%. The first one corresponds to those with relatively high lethality mutations, and the latter corresponds to the normal z/z homozygotes. Note that because *β*/*β* is lethal, only *β*/z and z/z genotypes can potentially survive. If the genotypes are of the same fitness, their proportions will be 2/3 and 1/3, respectively. Figure 3 shows that z/z appears to be less fit than the *β*/z genotype, resulting in an average of approximately 17.5% only. One possible reason may be that the normal z chromosome used in the initial experiment contained some mildly deleterious recessive mutations. The reduced fitness of the z/z homozygotes does not affect significantly the identification of recessive mutations of high lethality but makes the dissection of those of lower lethality more challenging.

The identification of mutations starts with lines having a low percentage of z/z offspring in the *F*_3_ and proceeds with cross experiments and inference. One example is presented to illustrate the process. Table 1 shows an example of the *F*_3_ results of a family early in the experiment. The family starts with 30 lines but after initial quality control (one with *β*/*γ*offspring and two with too few total offspring and a few lines did not survive), a total of 17 lines are recorded.

**Table 1:** An example of F3 data for family 140

Line #	*z*/*z*	Total	*z*/*z* Percent
1	0	110	0.000
2	3	90	0.033
3	0	56	0.000
5	29	112	0.259
6	2	72	0.028
9	3	127	0.024
11	0	120	0.000
12	1	64	0.016
14	1	101	0.010
15	0	70	0.000
16	2	63	0.032
18	3	71	0.042
19	1	54	0.019
24	2	103	0.019
28	0	62	0.000
29	1	125	0.008
30	1	118	0.008

It is clear that all lines, except for line 5, are suspects of new mutations of relatively high lethality. For a line to be declared as a mutant line for lethality level *d*, at minimum it should have a percentage of z/z offspring not larger than 1 − *d* and it is crossed with at least one other line if available. We were not sure how low the *d* can be so it was decided to perform as many crosses as feasible. As a result, all the lines except 5 and 2 are used for cross experiments. The exclusion of line 2 was not intentional but was due to the late completion of *F*_3_ for that line.

**Figure 4:**
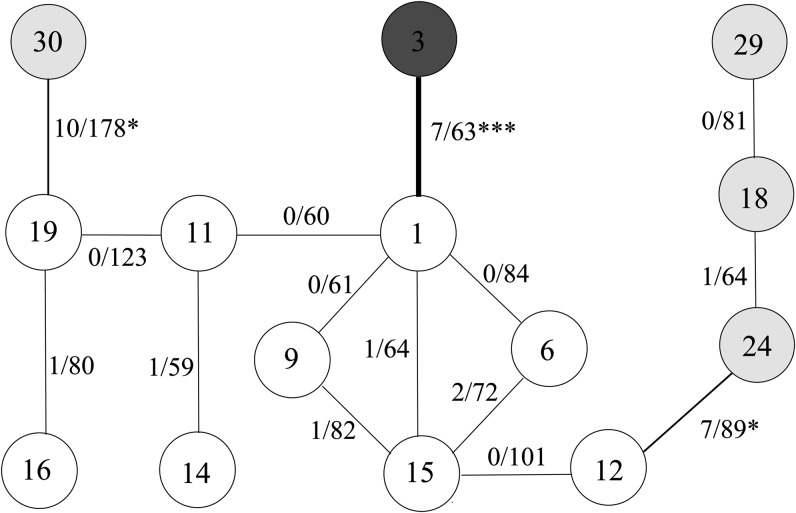
Cross and results for family 140. *a*/*b* (e.g. 0/101) beside a line indicates the numbers of z/z and total offspring of the cross, respectively. Three crosses, between lines 19 and 30, between 12 and 24, and between lines 1 and 3 are individually significant (with * and *** representing, respectively, significance at 5 and 1% level). After adjusting for multiple tests, all three crosses are significant at the 50% significance level but only the cross between lines 1 and 3 remains significant at the 10% significance level.

There are multiple ways to carry out allelism tests but the principle is that ambiguity needs to be resolved. Figure 4 shows the diagram of crosses used and their results. It is clear that there is no evidence that the six lines, represented by white circles, resulted from different mutations because the percentages of z/z offspring from crosses among them are similar to their parental lines. The crosses between lines 1 and 3, lines 19 and 30, and lines 12 and 24, resulted in significant test results, but after adjusting multiple tests as described in the *Statistical Method* section, only the cross between 1 and 3 is significant at the 10% level. Therefore, two mutations are identified, the first one includes line 3 with the z/z percentage being 0, and the second one includes the other 13 lines tested with an overall z/z percentage of 0.0171.

**Figure 5:**
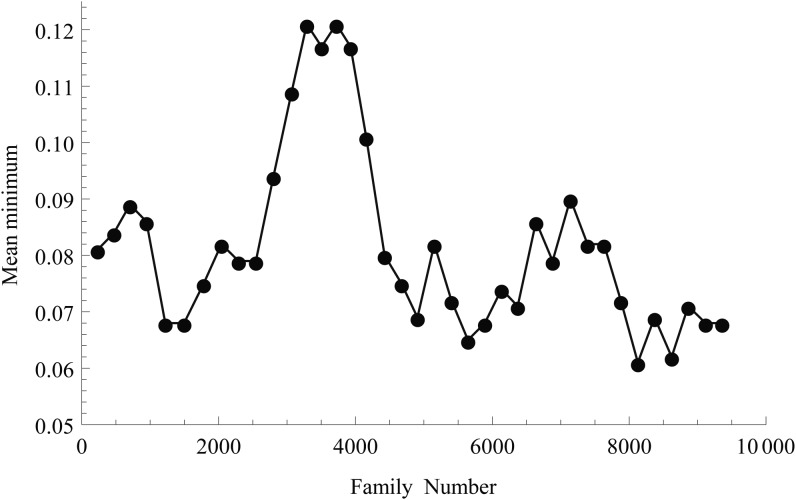
Mean minimum percentage of z/z offspring among nontested lines in windows of 200 families.

The number of identifiable mutations of relatively high lethality depends on the extensiveness of lines used in the allelism test. Although we were interested in determining as many mutations as feasible, it becomes too laborious when the number of lines to be crossed is large. Therefore, a compromise had to be made, which was achieved progressively. Figure 5 shows the mean minimum z/z percentage for lines in a family that are not subjected to the allelism crossing experiment. It can be seen that after the initial 3000 families, the minimum was raised from approximately 8% to approximately 12% for a short period of time. The practice was deemed impractical, and the minimum then dropped back to approximately 7%. The overall average is 8.2%.

Despite the fact that lines with a recessive lethality of more than 92% were generally subjected to the allelism test, there is an increasing level of difficulties and ambiguity in determining the identity of mutations with decreasing lethality. As a result, we are cautious about those mutations identified with a recessive lethality lower than 95%. To be conservative, yet allowing for sufficient contrast, we will focus on those with lethality of 97% or greater. The mutation patterns identified through the allelism test depends on how stringent the criteria is, which is controlled by the value of *α* as described in the *Materials and Methods* section. Because *α* is the overall error rate, the larger it is, there will be more independent mutations declared. Table 2 provides the summary of mutations identified under different lethality intervals under two values of *α*. Both the number of mutations and the number of mutants are greatest in the lethality interval [97%, 98%), followed by the lethality interval [98%, 99%). Overall, the pattern appears to agree with that of the z/z percentages shown in Figure 3, where the second percentile is the most frequent mutant type and is followed by the third. As predicted previously, the number of mutations under *α* = 0.5 is larger in most cases, particularly for greater lethalities. However, the overall mutation rate does not change significantly in any of the cases. It turns out that subsequent analysis of mutations from different *α* values also leads to a relatively small difference. Therefore, we will focus on the presentation of analysis resulting from the mutations identified with *α* = 0.10.

**Table 2:** Distributions of 9594 families by mutation count under different lethality intervals

Mutations	[99–100%]	[98–99%)	[97–98%)
Allelism test with *α* = 0.1			
0	8459	8391	8296
1	1052	1134	1240
2	81	66	56
3	2	3	2
* m_t_*	1220	1275	1358
* n_m_*	2673	10,766	17,342
$\tilde{\mu }$	0.0098	0.0396	0.0638
Allelism test with *α* = 0.5			
0	8304	8273	8265
1	1174	1219	1232
2	112	95	93
3	4	7	4
* m_t_*	1410	1430	1430
* n_m_*	2897	11,328	16,574
$\tilde{\mu }$	0.0107	0.0417	0.0610

*m_t_*, total number of mutations; *n_m_*, total number of mutants; $\tilde{\mu }$, overall mutation rate estimated by Equation (6).

The data analyzed by Gao *et al.* (2011) is a subset of those with lethality [99%,100%] (or ≥99%). However, a 5% significance level without adjustment for multiple tests was used, so their data are not strictly a subset of column 1 of Table 2 from either *α* = 0.10 or *α* = 0.50. Because the number of crosses in a family increases with the number of likely mutants, the major effect of adjusting for multiple tests is that it makes the declaration of different mutations easier for families with a smaller number of likely mutants and harder for families with many likely mutants. Comparatively, because the mean number of crosses for families with a cross is approximately 17 (data not shown), this translates into about a 3% significance level on average per family, even *α* = 0.5 still appears slightly more stringent than the criteria used by Gao *et al.* (2011). It is reassuring that the subsequent inference is rather robust and as a result no qualitative conclusions made previously need to be revoked, which will be seen from the inference in a later section.

It is also useful to consider mutations for given minimum lethality levels. Table 3 provides the distribution under four minimum lethalities (notice that lethality ≥99% is the same as [99%, 100%]). It should be pointed out that mutation patterns for a given minimum lethality, say 97%, cannot be obtained as a simple aggregation of those in the lethality intervals [99–100%], [98–99%), and [97–98%). This occurs because sometimes mutations of lethalities falling into different intervals may occur in the same family, which can be seen from Table 3 in which there are families with four mutations, whereas there are none when considering lethality intervals separately.

**Table 3:** Distributions of 9594 families by mutation count under different minimum lethalities

Mutations	≥99%	≥98%	≥97%
0	8459	7397	6321
1	1052	1919	2743
2	81	258	481
3	2	20	48
4	0	0	1
*m_t_*	1220	2495	3853
*n_m_*	2673	13439	30,781
$\tilde{\mu }$	0.0098	0.0494	0.1133

*m_t_*, total number of mutations; *n_m_*, total number of mutants; $\tilde{\mu }$, overall mutation rate estimated by Equation (6).

There are 111 families of size 20 (Figure 2) and under the lethality level [99%,100%] the collection of mutation patterns is

$$\mathrm{20}:{\langle 1\rangle }_{11}\langle 2\rangle \langle 3\rangle \langle 17\rangle {\langle 1,1\rangle }_{2}$$

which indicates that 11 have one mutation of pattern <1>, one each for patterns of <2>, <3>, and <17>, respectively and two have a mutation of pattern <1, 1>. The number of mutant families is 11 + 1 + 1 + 1 + 2 = 16 and, thus, the number of nonmutant families is 111 − 16 = 95. Similarly, the mutation patterns for a family of size 20 under lethality level [98%, 99%) and [97%,98%) are, respectively,

$$\mathrm{20}:{\langle 1\rangle }_{6}{\langle 2\rangle }_{2}{\langle 3\rangle }_{2}$$

and

$$\mathrm{20}:{\langle 1\rangle }_{8}\langle 2\rangle {\langle 3\rangle }_{2}\langle 14\rangle \langle 19\rangle \langle 2,10\rangle .$$

The complete mutation patterns of the data corresponding to different lethality levels are given Table A1, Table A2, and Table A3 in the Appendix. For the sake of space, mutation patterns for given minimum lethalities will not be listed but can be obtained from the authors.

### Inference about mutation rates

Although it is desirable to make an inference about the mutation rate for every cell division along germline development, the lack of sufficient resolution in the experimental data and the prohibiting computational burden limit our inference to six or fewer intervals. As described in the *Statistical Method* section, the intervals can be represented by a series of integers defining the boundary locations. For example, the sequence 1, 2, 14, and 31 means that there are five intervals: [1, 1], [2, 2], [3, 14], [15, 31], and [32, *n*] where *n* is the last cell division (*e.g.*, 36). However, because different sperm may experience different numbers of divisions (but at least 36), while regardless of the number, the last five are spermatogenesis, it is more logical to put the last five divisions into the last interval and divisions from the 15th to just prior to spermatogenesis as the fourth. This interval definitions is conveniently represented by 1, 2, 14, −5.

The evaluation of the likelihood function makes use of coefficients computed from simulated samples from the germline population, which is dependent on the assumptions about the population dynamics. Let *N*(*i*) be the population size of germline lineage after the *i*th cell division. The assumptions about *N*(*i*) used in Gao *et al.* (2011) are provided in Table 1 in Fu (2013) and one assumption that has significant impact on the analysis is about *N*(8). Previous knowledge (Drost and Lee 1995, 1998) suggests that after the eighth division, about four to six cells move to the posterior region and become the primordial germ cells (PGCs). For a relatively small number of PGCs, are the PGCs a random sample out of the 256 cells available after the eighth division, or are they more closely related being descendants of an ancestral cell a few divisions earlier, or something in between? We will refer to this factor as sampling bias leading to N(8). This issue can be investigated effectively by examining the value of *N*(5), because after three divisions a cell in N(5) will have eight descendant cells in N(8), making it possible that all the PGCs are derived from a single cell in N(5). On the other hand, if N(5) is set to 32, it is equivalent to assuming that N(8) is a random sample out of the 356 cells.

The maximum number *D* of cell divisions is one factor that impacts the likelihood analysis. Because 36 cell divisions is the minimum and young mature males were used in the experiment, D was set to 36 as in Gao *et al.* (2011), although large values also were examined to some extent. As pointed out in the *Experiment* section, the likely range of cell divisions for sperm leading to *F*_1_ offspring is between 36 to about 40. Hence, we conducted likelihood analysis with *D* from 36 to 42, and found that *D* = 38 yields the overall best fit, which is significantly better than the case with *D* = 36, but goodness of fit declines gradually after *D* > 40. Even so, the pattern of likelihood estimates differ only marginally for D in the range between 36 and 42 and the impact on the interpretation of inference is relatively low. Because we are reasonably confident that the majority of sperm used in the experiments are from young males, we shall report results throughout the paper with *D* = 38.

### Assumptions on the dynamics of the germline population

Although we are primarily interested in the distribution of mutation rates during germline development, it is important to evaluate the impact of the assumptions about the dynamics of the population on the inference. One such assumption concerns how the four to six cells are selected after the eighth cell division. The primary analysis in Gao *et al.* (2011) assumed that these cells are randomly drawn from 256 cells which resulted from the eighth cell division. Although this does seem consistent with the Drosophila embryonic development literature (Sonnenblick 1965), the impact of biased sampling were evaluated to some extent. We carried out more extensive analysis using the full data set.

We first investigated the impact of the values of *N*(5) and *N*(8) on the value of the likelihood function. To make the computation manageable, we divided the range (1−32) of N(5) into groups each containing two consecutive integers, and similarly the range (1−256) of *N*(8) into groups each containing three consecutive integers. Because the dynamics of the germline population are properties of the germline and thus should be more or less independent of types of mutations, one uses as much data as possible for judging its goodness of fit. Therefore, we examine the effect of gradually adding more data into the analysis on the strength of conclusions. Table 4 shows the log-likelihood values for a number of combinations of *N*(5) and *N*(8) for data under three lethality levels.

**Table 4:** Differential decrease to the maximum likelihood with intervals [1, 1], [2, 2], [3, 14], [15 − 6], [−5, 38]

		Recessive Lethality		
*N*(5)	*N*(8)	≥99%	≥98%	≥97%
1−2	1−3	80.3	622.9	1519.1
	4−6	31.7	261.6	636.8
	7−9	42.5	329.3	809.6
	10−12	53.4	403.2	996.1
	13−15	59.6	443.3	1093.4
3-4	1−3	53.6	396.8	958.7
	4−6	5.3	48.9	114.9
	7−9	2.2	26.0	55.9
	10−12	2.8	27.3	62.6
	13−15	3.8	32.7	75.3
5-6	1−3	54.2	401.8	970.8
	4−6	2.1	20.8	47.5
	7−9	0.0	0.0	0.0
	10−12	1.0	3.3	13.6
	13−15	2.0	8.8	30.6
7-8	1−3	53.3	401.8	963.3
	4−6	2.3	17.7	39.3
	7−9	1.9	7.1	25.3
	10−12	4.0	17.3	56.6
	13−15	5.7	27.1	84.7
9-10	1−3	52.5	398.1	953.3
	4−6	2.8	18.3	45.4
	7−9	3.7	16.6	53.4
	10−12	6.7	32.8	100.3
	13−15	9.2	46.7	128.9

Table 4 shows that regardless of recessive lethality the maximum likelihood is achieved at the combination of *N*(5) = 5 ~ 6 and *N*(8) = 7 ~ 9. Because twice the difference is the value of the log-likelihood ratio test (with one degree of freedom when one of the *N*(5) and *N*(8) is fixed), it follows that for 98% and 97% recessive lethality, any other combination of *N*(5) and *N*(8) can be rejected at the 1% significance level. For 99% recessive lethality, the resolution is less, but all other combinations except *N*(5) = 5 ~ 6 and *N*(8) = 10 ~ 12 can be rejected at the 5% level (including the random sampling). These results imply that *N*(5) is about 5−6, which represents a sampling for the eighth population that is far more restrictive than random sampling, which corresponds to *N*(5) = 32. Note that Gao *et al.* (2011) categorically defined *N*(5) = 4 as mild sampling bias largely due to mistaking the value as the number of ancestral sequences after the fifth cell division. Given the amount of reduction from 32 to 5−6, its classification as modest bias (at least) appears to be in order. These results also suggest that *N*(8) = 4 − 6, based on various experimental observations, appears to be conservative. Hence, a slightly larger number for *N*(8) may be more the norm.

### Estimates of mutation rates

In the previous section, we established that *N*(5) = 5 ~ 6 and *N*(8) = 7 ~ 9 is the best assumption for the germline population dynamics; therefore, we will use this assumption for subsequent analysis. Because we investigate the mutation rates for different lethality levels, it is desirable to know whether a slight deviation of the optimal parameters will lead to a significant difference in the subsequent mutation rate estimation and hypothesis testing. We performed full likelihood analysis for several lethalities around *N*(5) = 5 − 6 and *N*(8) = 7 − 9 and found that indeed the impact is rather marginal unless the deviation leads to a substantially smaller likelihood value. Table 5 lists, as examples, the maximum likelihood estimates of mutation rates for combinations of *N*(5) and *N*(8) that differs no more than one step from the optimal. As can be seen, overall the estimates are rather similar to those under the optimal, but there are some notable differences in *u*_2_ for cases two steps away from the optimal; but then the difference of likelihood to that of the optimal is substantial. Therefore, we are confident that conclusions based on the optimal *N*(5) and *N*(8) will be robust.

**Table 5:** Full maximum likelihood estimates of *u* × 10^3^ for lethality ≥ 97% and *N*(5) = 5 ~ 6 and *N*(8) = 7 ~ 9 with intervals [1,1], [2,2],[3,3], [4 14], [15 -6], and [-5 38]

*N*(5)	*N*(8)	*u*_1_	*u*_2_	*u*_3_	*u*_4_	*u*_5_	*u*_6_	−*ln*(*L*)
3−4	4−6	68.666	5.685	0.000	0.001	0.189	1.955	15272.36
3−4	7−9	69.001	3.028	0.000	0.001	0.190	1.951	15216.65
3−4	10−12	68.801	2.388	0.000	0.001	0.193	1.951	15226.09
5−6	4−6	68.801	2.393	0.001	0.001	0.190	1.951	15211.19
5-6	7−9	67.082	1.635	0.000	0.002	0.187	1.951	15164.41
5−6	10−12	66.437	1.374	0.013	0.002	0.188	1.951	15175.13
7−8	4−6	67.866	1.876	0.000	0.001	0.191	1.951	15205.74
7−8	7−9	65.919	1.316	0.013	0.002	0.189	1.951	15185.64
7−8	10−12	65.216	1.167	0.000	0.002	0.190	1.951	15213.69

Table 6 gives the estimates of ***u*** for three recessive lethalities for 38 cell divisions and the optimal combination of *N*(5) and *N*(8). The most striking pattern is that the mutation rate for the first division varies considerably, but regardless of how the data are examined, it is markedly larger than those for subsequent divisions. Similar to the pattern observed in Gao *et al.* (2011), the spermatogenesis has a relatively higher mutation rate than the interval divisions. With data aggregation, there appears to be a trend of appreciable mutation rate for the second division, and a similar pattern is observed for the stem cell stage although the magnitude is less appreciable. The cleavage stage, excluding the first (and perhaps also the second), harbors the smallest mutation rate. The ratio of the first division mutation rate to the mean mutation rate of the internal divisions are all larger than 100, with the highest ratio of 620 for lethality [97%, 98%).

**Table 6:** Full maximum likelihood estimates of *u* × 10^3^ for several lethalities

Lethality	*u*_1_	*u*_2_	*u*_3_	*u*_4_	*u*_5_	*u*_6_	$\hat{\mu }$	*Ratio*
[99%, 10%)	4.054	0.000	0.000	0.001	0.028	0.850	0.0089	206
[98%, 99%)	24.460	0.337	0.006	0.010	0.067	0.551	0.0289	469
[97%, 98%)	39.577	0.605	0.002	0.002	0.077	0.398	0.0437	620
≥ 98%	28.660	0.272	0.006	0.018	0.089	1.443	0.0380	439
≥ 97%	67.371	1.258	0.021	0.031	0.177	1.954	0.0821	445

Ratio: the ratio of the mutation rate of the first cell division and the mean rate for the interval cell divisions, computed as u1/[(u2 + u3 + 11u4 + 18u5)/32].

### Mutation rate hypotheses testing

As defined in *Materials and Methods*, ***μ*** = (*u*_1_, *u*_2_, … *u_I_*) with *u_i_* being the mutation rate per cell division in the *i*th interval. Then different constraints on the rates will affect the estimates of ***μ*** and the associated log-likelihood values are used as the basis for testing the hypotheses about the mutation rates. The following nine hypotheses will be considered:

$$H_{1}:u_{1}=\ldots .=u_{I}\left(\text{rates}\;\text{are}\;\text{constant}\right)$$

$$H_{2}:u_{2}=\ldots =u_{I-1}\left(\text{rates}\;\text{for}\;\text{all}\;\text{intervals}\;\text{except}\;\text{the}\;\text{first}\;\text{and}\;\text{last}\;\text{are}\;\text{equal}\right)$$

$$H_{3}:u_{1}=u_{2}\left(\text{rates}\;\text{for}\;\text{the}\;\text{first}\;\text{two}\;\text{intervals}\;\text{are}\;\text{equal}\right)$$

$$H_{4}:u_{2}=u_{3}\left(\text{rates}\;\text{for}\;\text{the}\;\text{second}\;\text{and}\;\text{third}\;\text{are}\;\text{equal}\right)$$

$$H_{4b}:u_{3}=u_{4}\left(\text{rates}\;\text{for}\;\text{the}\;\text{third}\;\text{and}\;\text{fourth}\;\text{are}\;\text{the}\;\text{same}\right)$$

$$H_{5}:u_{I-2}=u_{I-1}\left(\text{rates}\;\text{for}\;\text{the}\;\text{second}\;\text{and}\;\text{third}\;\text{interval}\;\text{to}\;\text{the}\;\text{last}\;\text{are}\;\text{equal}\right)$$

$$H_{6}:u_{I-1}=u_{I}\left(\text{rates}\;\text{for}\;\text{the}\;\text{last}\;\text{two}\;\text{intervals}\;\text{are}\;\text{equal}\right)$$

$$H_{7}:u_{1}=u_{I}\left(\text{rates}\;\text{for}\;\text{the}\;\text{first}\;\text{and}\;\text{last}\;\text{are}\;\text{equal}\right)$$

$$H_{8}:\text{no}\;\text{restriction}.$$

All the hypotheses except *H*_4_*_b_* are labeled the same as those in Gao *et al.* (2011) and Fu (2013). Table 7 gives the values of these tests against the *H*_8_.

**Table 7:** The values of the log-likelihood ratio test for various hypotheses *H_i_* against *H*_8_

	*i*							
Lethality	1	2	3	4	4b	5	6	7
[98%, 99%)	2304.9	1.0	176.3	0.5	0.4	0.7	236.1	455.9
[97%, 98%)	4197.6	3.6	345.7	1.0	0.0	3.4	142.7	893.0
≥99%	1004.3	3.1	31.9	0.0	0.0	2.5	654.5	52.2
≥98%	2953.9	1.2	193.0	0.2	0.2	0.6	855.6	492.3
≥97%	6613.4	1.6	489.7	0.9	1.1	1.3	934.3	1287.6

Asymptotic *χ*^2^ distribution for *H_i_* against *H*_8_ has 4, 2, 1,1,1,1 and 1 degree of freedom, respectively.

As in Gao *et al.* (2011), the assumption of equal mutation rate during germline development is soundly rejected and the evidence is stronger with data aggregation. As mentioned previously, with data aggregation there appears to be a trend that the second division and the stem-cell stage also have appreciable mutations (Table 6), but the log-likelihood ratio tests in Table 7 does not provide significant support for the trend. In fact, there is no significant evidence to reject the hypothesis that all internal divisions, that is, from the second until right before gametogenesis, share the same mutation rate.

To investigate the sensitivity of our inference with regard to possible sporadic inclusions of families with pre-existing mutations, we can exclude a certain fraction of families with a high percentages of mutants among offspring. However, it is not easy to decide the proper fractions to use, so a conservative approach was taken by removing all families with a percentage of mutants exceeding a given threshold value. Table 8 shows the results of the likelihood ratio tests for two different thresholds. At a 90% threshold value, for example, families of size 20 with 18 or more mutants were excluded, and families of size 30 with 27 or more mutants were excluded. As expected, the number of families excluded increases with the width of the lethality interval for a given threshold value, and decreasing the threshold from 90% to 85% results in doubling the number of families being removed. For the latter threshold value, as high as 20% of mutant families were removed. Even with such extreme exclusions, all the cases with significant test results shown in Table 7 remain the same.

**Table 8:** The values of the log-likelihood ratio test for various hypotheses *H_i_* against *H*_8_ excluding families with high percentage of mutants

		*i*							
Lethality	No. Excluded Families	1	2	3	4	4b	5	6	7
Mutants % ≥ 90									
≥99%	22	1020.2	2.3	12.4	0.0	0.1	0.7	658.8	15.3
≥98%	125	2155.2	0.7	94.0	1.0	0.0	1.9	843.5	228.3
≥97%	324	4322.4	0.0	219.6	7.8	3.2	15.3	934.3	613.4
Mutants% ≥ 85									
≥99%	43	1007.5	0.6	6.2	0.0	0.0	0.6	660.7	5.3
≥98%	272	1792.2	0.6	55.9	1.2	0.0	1.5	835.9	130.7
≥97%	665	3283.6	4.5	125.7	7.9	0.0	8.2	921.8	339.7

Asymptotic *χ*^2^ distribution for *H_i_* against *H*_8_ has 4, 2, 1, 1, 1, 1, and 1 degree of freedom, respectively.

So far we have grouped the five cell divisions in the spermatogenesis as one interval and thus assumed that mutation rates are constant within the interval. This hypothesis can be investigated by dividing the gametogenesis into two intervals. Table 9 lists the maximum likelihood estimates for different partitions of the five cell divisions in gametogenesis (to reduce the amount of computation, the third cell division and the remaining cleavage divisions are combined into one interval, since there is little evidence to suggest that their rates are different). It follows that the maximum difference in the log-likelihood value to that for the case of equal rate (i = 0) is 0.7. Therefore, there is little evidence to suggest different mutation rates in the process of gametogenesis, despite the trend that early cell divisions have a greater estimated mutation rate than the later divisions.

**Table 9:** Likelihood estimates of mutation rates with lethality ≥97% with gametogenesis split into two intervals with [1, 1], [2, 2], [3, 14], [15 − 6][−5, −(*i* + 1)], [−*i*, 38]

i	*u*_1_	*u*_2_	*u*_3_	*u*_4_	*u*_5_	*u*_6_	−*ln*(*L*)
0	67.082	1.457	0.016	0.183	1.936	1.936	15,164.7
1	67.082	1.425	0.023	0.165	2.282	0.825	15,164.0
2	67.082	1.379	0.026	0.167	2.279	1.548	15,164.1
3	67.082	1.289	0.034	0.162	2.429	1.713	15,164.3
4	67.082	1.240	0.039	0.159	2.814	1.797	15,164.1

*i*/(5 − *i*): first *i* divisions of gametogenesis and last 5 − *i* divisions as two intervals.

## Discussion

The full data set from our large-scale mutation screening experiment provides rich information for exploration in much more detail than previously possible for various aspects of the mutation process during germline development. The inference, taking advantage of the improved framework, leads to the following conclusions: (1) mutation rates during germline development are not equal, (2) the first division harbors the greatest mutation rate, (3) gametogenesis has mutation rates greater than other divisions except the first (and perhaps the second as well), (4) after the first cell division, the rate drops rapidly, and after the second division the rate becomes flat throughout the cleavage stage, (5) no evidence of rate difference during the gametogenesis, and (6) the number of PGCs after the eighth division is likely greater than that reported in the literature and they are not derived at random from the 256 cells at that stage nor from one or two ancestral cells at the fifth division.

Because of reduced DNA repair efficiency during gametogenesis, greater mutation rate at gametogenesis is expected. Although it is generally known that zygotic control starts toward the end of the cleavage stage which for *Drosophila* is around the 10−14th divisions, current knowledge of *Drosophila* development does not provide an adequate explanation of why the first division (or with the second division) harbors a much elevated mutation rate compared with the rest of the cell divisions during the cleavage stage, which was pointed out earlier by Gao *et al.* (2011). However, it is now known that some mutations in the sperm may be repaired in the early cleavage after fertilization (Rathke *et al.* 2014), so a portion of observed mutations in the early cleavage may be the result of incomplete repairing of pre-existing mutations. If all early cleavage mutations are derived as such, one would expect that the mutation rate for the first cell division would be similar to or smaller than that of gametogenesis; therefore, the much elevated mutation rate for the first cell division remains to be illuminated biologically.

To safeguard our conclusions against artifacts in both the experiment and inference, we also carried out analysis with combinations of parameters deviated from the reported optimal set. Examining the impact of assumptions on the dynamics of population size is one such effort, another is to examine the impact of sporadic pre-existing mutations of high lethality that had managed to escape our surveillance. Such mutations would be identified by the experiment as ones that lead to 100% mutant offspring (if the experiment was perfect) or close to 100% mutant offspring if the z/z percentages in a few lines fluctuate upward to escape both the initial screening and subsequent allelism tests. Our analysis (Table 8) shows that the aggressive removal of families with a high percentage of mutants does not change the main conclusions. Therefore, the possibility of some sporadic pre-existing mutations escaping our surveillance cannot be the primary cause of the sharp contrast of mutation rates along the germline development. The experiment and subsequent dissection of mutations was not perfect, which led to our investigation of the impact of the overall significance level on assigning mutants into mutational groups and subsequent inference. Again, all the main conclusions remains intact. One can conclude that the consistency of conclusions under various adjustments come from overwhelming information (both quantity and quality) in the data.

The greater resolutions in data from 98% and 97% recessive lethality have resolved some ambiguity previously encountered but also reveals a striking pattern. Although the rate for gametogenesis increases from 1 to 1.5 and 1.9, the rate increase of the first cell division is much more profound, from 4 to 30 for 98% lethality and 60 for 97% lethality. Although some bias might have been introduced in the delineation of mutations from cross data, the impact of such factors is likely modest at best. This is because the same analysis was conducted for mutation patterns generated by using two different *α* values (0.5 and 0.95) and the conclusions remain mostly intact except for some relatively small changes in numerical values. Therefore, we are confident that the elevated mutation rate for the first cleavage with decreasing lethality is beyond reasonable doubt. However, there is an increasing discrepancy between the overall mutation rate estimated from likelihood analysis and that from the classical estimator when the rate is significantly higher than 1%. We are not certain about the cause of this discrepancy but again different criteria for delineating cross data are not the major cause. This is an issue that deserves further investigation and one possible reason may be due to increasing inaccuracy of approximating the larger probabilities of mutation patterns used in the likelihood analysis. If this is true, it implies that the mutation rate for the first cell division might have been underestimated. Nevertheless, the qualitative conclusions from the current analysis are unlikely to be altered significantly.

The pattern of the ratio of the mutation rate of the first cell division and the mean rate of the interval divisions also deserve to be examined carefully. The lower ratio for greater lethality level (≥99%) than those of lower lethality, say [97%,98%) for example, is apparent. One possible cause may be that a significant number of mutations, which are completely lethal or nearly lethal, have a dominant effect and the degree of dominance increases with the lethality in general. When the lethality is reduced, the second division starts to harbor a greater mutation rate, which also helps to lower the ratio. Another way to look at the phenomenon is to examine the average cluster size of mutations for different levels of lethality. Table 2 shows that on average (*i.e.*, *n_m_*/*m_t_*), mutations of lethality ≥ 99 is 2.2 whereas the average for lethalities of level [98%, 99%) and [97%, 98%) are 8.4 and 12.8, respectively. In general, mutations leading to smaller clusters tend to occur later than those to larger clusters and thus the selective disadvantage of mutations of high lethality may be one reason which prevents them from reaching a high frequency in the cell population within a host.

It is clear that our experimental data have the resolution to test the validity of some assumptions about the dynamics of the population. The number of cell divisions for the sperm in the parental stage was initially thought to be 36, but after examining the experimental procedure carefully, we realized that by the time the *β*/z males were introduced into the parental stage, additional two to three stem cell divisions might have occurred because on average it takes 32 hr for a stem-cell cycle (Wallenfang *et al.* 2006). Furthermore, each male can only mate a limited number of times during 24 hr; thus, the offspring in the *F*_1_ may result from sperm of an even wider range of ages. Therefore, sperm in the Parental stage likely range from a minimal of 36 cell divisions to approximately 40 divisions. Indeed, the likelihood analysis suggests that 38 cell divisions provide the best overall fit. The population size after the eighth division was thought to be in the range of four to six as reported in the literature (Drost and Lee 1995, 1998), but the best fit suggests that may be a slight underestimate, and a more appropriate range should be 7−9. Although the aforementioned two quantities have previous knowledge to judge their validity, little can be found from the literature about how the PGCs after the eighth division are derived or selected among the 256 available cells. Constraints of physical space and the tendency that recently derived cells tend to cluster near each other suggests that the PGCs may not be a random sample from the 256 cells as assumed by default. This analysis provides strong evidence that they are derived from a relatively small number of ancestral cells at the fifth division, but the assumption that their common ancestor is a cell at the fifth generation can be soundly rejected.

Regardless of the mechanism leading to the increased mutation rate ratio with decreasing lethality between the first division and gametogenesis, the trend is clear, and one can envisage that for neutral or nearly neutral mutations, the ratio may be even greater. This prospect lends stronger support to the explanation in Gao *et al.* (2011) concerning a lower ratio of male *vs.* female mutation rate than expected due to the difference in the number of cell divisions between sperm and eggs. This study further suggests that the rate differential between the first one or two with the remaining cell divisions can differ for mutations of different nature (here different lethalities). It is conceivable that this may be true for different genes/regions in the genome as well. Human genetic disease cases (Vogel and Motulsky 1997) as well as DNA sequence data (Miyata *et al.* 1987; Shimmin *et al.* 1993; Li 1997; Crow 2006) has led to the conclusion known as male-driven evolution, *i.e.*, males dominate females in generating inheritable mutations in evolution. For 30-yr-old human males and females, there are roughly 400 and 30 cell divisions leading to sperm and eggs, respectively (Drost and Lee 1995), so the expected ratio of male-to-female mutation rate is larger than 10; however, estimates from sequence data vary and in general are lower than this ratio. Several possible causes have been put forward for this apparent discrepancy, but if the first (or first and second) cell division has a mutation rate many-fold larger than the average mutation rates for the remaining cell division, the phenomenon becomes easily explainable. For example, suppose the ratio of the mutation rate of the first cell division and the mean of the internal divisions is 100, then the male to female ratio would be expected to be (100 + 399)/(100 + 29) ≈ 3.9 and if the ratio is 500, then the male to female mutation ratio would be about 1.7. Because different mutation types or regions may exhibit rather different ratios between the first division and the average, the ratio between male-to-female mutation rates can be expected to be quite variable.

## Appendix

### Mutation patterns for different recessive lethalities

**Table A1:** Mutation patterns for recessive lethality [99–100%)

**2**: <2> **3**: <1>_6_ **4**: <1>_4_ < 3><1, 1> **5**: <1>_9_ < 3> **6**: <1>_9_ < 2> <1, 1>_2_ **7**: <1>_4_ < 6> **8**: <1>_9_ < 3> **9**: <1>_6_ < 2><3> **10**: <1>_12_ < 2>_4_ < 4> **11**: <1>_8_ < 3><2, 5> **12**: <1>_3_ < 3><1, 6> **13**: <1>_5_ < 2>_2_ < 3><1, 1> **14**: <1>_8_ < 1, 3> **15**: <1>_7_ < 1, 1> **16**: <1>_5_ < 3> **17**: <1>_7_ < 1, 1> **18**: <1>_9_ **19**: <1>_10_ **20**: <1>_11_ < 2><3><17><1, 1>_2_ **21**: <1>_19_ < 2><1, 1> **22**: <1>_21_ < 2>_3_ < 1, 1><1, 2> **23**: <1>_22_ **24**: <1>_24_ < 4><1, 1>_3_ **25**: <1>_21_ < 1, 1>_2_ **26**: <1>_26_ < 2><3>_2_ < 8><1, 1> **27**: <1>_36_ < 2>_4_ < 1, 1>_3_ < 1, 4, 19> **28**: <1>_38_ < 2><3><1, 1><1, 2><2, 5> **29**: <1>_32_ < 2><1, 1><1, 23> **30**: <1>_55_ < 2>_3_ < 25><27><29><1, 1>_2_ < 1, 3><1, 4><2, 3> **31**: <1>_76_ < 2>_3_ < 3><26>_2_ < 28><29><31><1, 1>_2_ < 1, 3> **32**: <1>_90_ < 2>_4_ < 3><26><27><29>_2_ < 30>_4_ < 1, 1>_7_ < 1, 16><1, 24><1, 26><1, 27><1, 1, 1> **33**: <1>_134_ < 2>_4_ < 3>_2_ < 21><25><26><27><30><32><33>_2_ < 1, 1>_12_ < 1, 2><1, 27><1, 28><3, 27><4, 25> **34**: <1>_131_ < 2>_4_ < 3>_5_ < 5><22><25>_2_ < 29>_2_ < 31>_2_ < 33><1, 1>_7_ < 1, 2><1, 3>_2_ **35**: <1>_86_ < 2><3><4><28><30>_2_ < 31><32><33><1, 1>_6_ < 1, 2>

**Table A2:** Mutation patterns for recessive lethality [98–99%)

**2**: <1>_3_ **3**: <1> **4**: <1>_4_ < 2> **5**: <1>_4_ **6**: <1>_6_ **7**: <1>_5_ < 2>_2_ **8**: <1>_2_ < 2> **9**: <1>_4_ < 2> **10**: <1>_8_ < 3><4> **11**: <1>_7_ < 2>_2_ **12**: <1>_5_ < 2><7> **13**: <1>_5_ < 3><4><7> **14**: <1>_6_ < 2>_2_ < 6> **15**: <1>_8_ < 2><3>_2_ < 5> **16**: <1>_6_ < 2><3><1, 1> **17**: <1>_5_ < 2>_3_ < 3><5><11><13><1, 1><1, 9> **18**: <1>_10_ < 2>_2_ < 4> **19**: <1>_6_ < 2>_4_ < 4><13> **20**: <1>_6_ < 2>_2_ < 3>_2_ **21**: <1>_11_ < 2><4> **22**: <1>_10_ < 2>_3_ < 3><10><11><1, 1> **23**: <1>_15_ < 2><3><9><17><21> **24**: <1>_22_ < 2>_2_ < 3><5><10><16><3, 17> **25**: <1>_16_ < 2><6><22> **26**: <1>_21_ < 2>_6_ < 9><18> **27**: <1>_18_ < 4><9><12><20><21> **28**: <1>_23_ < 2>_3_ < 3>_2_ < 6><7><18><21><1, 1>_3_ **29**: <1>_42_ < 2>_8_ < 14><16><20><21>_2_ < 23><25>_4_ < 1, 20><7, 10><11, 14><1, 1, 2> **30**: <1>_31_ < 2>_3_ < 3><4><6>_2_ < 7><15><17>_2_ < 19><20><22><25>_2_ < 26>_3_ < 27>_3_ < 28><29><1, 1>_4_ < 1, 2><1, 27><2, 5><4, 20><1, 1, 5> **31**: <1>_39_ < 2>_6_ < 3>_3_ < 4>_2_ < 5><9><10>_2_ < 19><22>_2_ < 23>_6_ < 24><25>_4_ < 26>_4_ < 27>_3_ < 28>_5_ < 29><30><1, 1>_2_ < 1, 25><6, 22><8, 9><2, 4, 15> **32**: <1>_64_ < 2>_8_ < 3><4>_2_ < 5><6><7><10><12><13><19><22><23>_2_ < 24>_2_ < 25>_6_ < 26>_9_ < 27>_4_ < 28>_3_ < 29>_8_ < 30>_3_ < 31>_4_ < 32><1, 1>_2_ < 1, 2>_2_ < 1, 13><1, 26><1, 28><2, 17><4, 18> **33**: <1>_69_ < 2>_15_ < 3>_4_ < 4><5>_2_ < 7><9><11><13>_2_ < 14><17><18><20><21><22>_3_ < 23><24>_4_ < 25>_5_ < 26>_9_ < 27>_7_ < 28>_10_ < 29>_9_ < 30>_12_ < 31>_6_ < 32>_6_ < 33>_2_ < 1, 1>_7_ < 1, 2><1, 3><1, 22><1, 27><1, 29><3, 23><3, 25><6, 19><6, 20><6, 23> **34**: <1>_93_ < 2>_5_ < 3><4><6><8><10><16><18><20><22>_3_ < 23>_3_ < 24>_3_ < 25><26>_3_ < 27>_5_ < 28>_12_ < 29>_9_ < 30>_12_ < 31>_10_ < 32>_7_ < 33>_3_ < 34>_4_ < 1, 1>_3_ < 1, 3><1, 20><1, 22><1, 25>_2_ < 1, 31><2, 26><2, 29><7, 21> **35**: <1>_67_ < 2>_8_ < 3>_2_ < 4><5>_2_ < 15>_2_ < 20><21><22>_3_ < 23>_2_ < 24>_2_ < 25>_2_ < 26>_6_ < 28>_3_ < 29>_5_ < 30>_6_ < 31>_10_ < 32>_2_ < 33>_3_ < 34>_3_ < 35><1, 1><1, 27><2, 2><3, 30>

**Table A3:** Mutation patterns for recessive lethality [97–98%)

**2**: <1> **3**: <1>_2_ **4**: <1>_2_ **5**: <1>_2_ **6**: <1>_4_ **7**: <1><4><6><1, 2> **8**: <1>_7_ < 2>_3_ < 2, 2> **9**: <2><3> **10**: <2>_2_ < 3> **11**: <1>_4_ < 2>_2_ < 3>_2_ < 6><9> **12**: <1> **13**: <1>_3_ < 2> **14**: <1>_2_ < 2>_2_ < 3> **15**: <1>_4_ < 3><7>_2_ < 12><13> **16**: <1>_4_ < 4><5> **17**: <1>_5_ < 2>_3_ < 3>_2_ < 13>_2_ < 17> **18**: <1>_4_ < 2><15> **19**: <1>_6_ < 2>_2_ < 3><4><11>_2_ < 2, 7> **20**: <1>_8_ < 2><3>_2_ < 14><19><2, 10> **21**: <1>_6_ < 3><8><12> **22**: <1>_13_ < 2>_5_ < 4><6><8><14> **23**: <1>_10_ < 2>_4_ < 4>_2_ < 11><12> **24**: <1>_12_ < 2>_4_ < 3><12><16><1, 1> **25**: <1>_16_ < 2><5>_2_ < 10><18> **26**: <1>_12_ < 2>_2_ < 4>_2_ < 11><16><17><21><23><1, 1><1, 2> **27**: <1>_19_ < 2>_3_ < 3><4><9><10><14><16><20><21><23>_2_ < 1, 6><2, 2> **28**: <1>_16_ < 2>_5_ < 3>_2_ < 4><6><9><10><20><21>_2_ < 25><27><1, 2> **29**: <1>_22_ < 2>_5_ < 3><7><20>_2_ < 21>_2_ < 22>_3_ < 23>_3_ < 25><27><28>_2_ < 1, 19><1, 24><2, 22> **30**: <1>_25_ < 2>_4_ < 4>_2_ < 6><16><17><18>_2_ < 19><20><23><24>_2_ < 25><26>_5_ < 27>_2_ < 28>_2_ < 29>_2_ < 1, 1><1, 9> **31**: <1>_35_ < 2>_10_ < 3>_2_ < 4><5>_3_ < 10><11><16><17>_2_ < 19>_3_ < 20>_2_ < 21>_3_ < 22>_3_ < 23>_3_ < 24>_4_ < 25>_2_ < 26>_2_ < 27>_8_ < 28>_6_ < 29>_6_ < 30>_5_ < 31><1, 1>_3_ < 1, 2><2, 23><2, 25><3, 17> **32**: <1>_61_ < 2>_10_ < 3>_6_ < 4><5><6><9><12><15><17><18><19><20>_2_ < 21>_4_ < 22>_3_ < 23>_3_ < 24>_4_ < 25>_7_ < 26>_11_ < 27>_4_ < 28>_7_ < 29>_12_ < 30>_6_ < 31>_7_ < 32>_3_ < 1, 1><1, 27><1, 29><3, 26><7, 24> **33**: <1>_59_ < 2>_9_ < 3>_5_ < 4>_3_ < 6><7><11>_3_ < 13><14>_2_ < 17><18><19>_3_ < 20>_5_ < 21>_5_ < 22>_2_ < 23>_10_ < 24>_7_ < 25>_8_ < 26>_8_ < 27>_17_ < 28>_15_ < 29>_12_ < 30>_13_ < 31>_16_ < 32>_18_ < 33>_6_ < 1, 1>_2_ < 1, 18><1, 25><1, 27>_2_ < 2, 20><3, 16>_2_ < 4, 25><6, 23> **34**: <1>_59_ < 2>_9_ < 3>_5_ < 5><8><10><12><14><16><17><19>_2_ < 20>_3_ < 21>_2_ < 22><23>_4_ < 24>_3_ < 25>_7_ < 26>_18_ < 27>_14_ < 28>_11_ < 29>_16_ < 30>_16_ < 31>_16_ < 32>_18_ < 33>_4_ < 34>_9_ < 1, 1>_2_ < 1, 2>_2_ < 1, 24>_2_ < 2, 27><3, 27><3, 29><6, 21><9, 18><10, 15>_2_ < 11, 13><1, 1, 1> **35**: <1>_51_ < 2>_4_ < 3>_5_ < 4>_2_ < 6><15><20><23><24>_3_ < 25>_2_ < 26>_4_ < 27>_5_ < 28>_7_ < 29>_9_ < 30>_11_ < 31>_7_ < 32>_12_ < 33>_8_ < 34>_4_ < 35>_3_ < 1, 2><1, 25><3, 28><9, 19><1, 1, 1>

## References

[bib1] Abrahamson S. Lewis E. B., 1971 The detection of mutations in *Drosophila melanogaster*, pp. 461–484 in Chemical Mutagens, edited by Hollaender A. Plenum Press, New York

[bib2] Ashburner M., 1989 Drosophila: A Laboratory Handbook. Cold Spring Harbor Laboratory, Cold Spring Harbor, NY

[bib3] Brodberg R. K. Mitchell M. J. Smith S. L. Woodruff R. C., 1987 Specific reduction of N-dimethylnitro-samine mutagenicity in *Drosophila melanogaster* by dimethyl sulfoxide. Environ. Mol. Mutagen. 10: 425–432 3119334 10.1002/em.2850100411

[bib4] Crow J. F., 2006 Age and sex effects on human mutation rates: an old problem with new complexities. J. Radiat. Res. 47 Suppl B**:** B75–82 17019055 10.1269/jrr.47.b75

[bib5] Drost J. B. Lee W. R., 1995 Biological basis of germline mutation: Comparisons of spontaneous germline mutation rates among *Drosophila*, mouse and human. Environ. Mol. Mutagen. 25(Suppl 26)**:** 48–64 7789362 10.1002/em.2850250609

[bib6] Drost J. B. Lee W. R., 1998 The developmental basis for the germline mosaicism in mouse and *Drosophila melanogaster*. Genetica 102/103: 421–443 9720293

[bib7] Fu Y. X., 2013 Statistical methods for analyzing *Drosophila* germline mutation rates. Genetics 194: 927–936 23636740 10.1534/genetics.113.151571 PMC3730920

[bib8] Fu Y. X. Huai H., 2003 Estimating mutation rate: how to count mutations? Genetics 164: 797–805 12807798 10.1093/genetics/164.2.797 PMC1462584

[bib9] Gao J. J. Pan X. R. Hu J. Ma L. Wu J. M., 2011 Highly variable recessive lethal or nearly lethal mutation rates during germline development of male *Drosophila melanogaster*. Proc. Natl. Acad. Sci. USA 108: 15914–15919 21890796 10.1073/pnas.1100233108 PMC3179084

[bib10] Gilbert S. F., 2003 Developmental Biology. Ed. 7 Sinauer Associates, Inc., Sunderland, MA

[bib11] Graf U. van Schaik N. Wurgler F. E., 1992 Drosophila Genetics: A Practical Course. Springer-Verlag, Berlin, Heidelberg

[bib12] Greenspan S. F., 1997 Fly Pushing: The Theory and Practice of Drosophila Genetics. Cold Spring Harbor Laboratory, Cold Spring Harbor, NY

[bib13] Huai H. Woodruff R. C., 1997 Clusters of identical new mutations can account for the overdispersed molecular clock. Genetics 147: 339–348 9286693 10.1093/genetics/147.1.339 PMC1208118

[bib14] Li W. H., 1997 Molecular Evolution. Sinauer Associates, Inc., Sunderland, MA

[bib15] Lindsley D. L. Zimm G. G., 1992 The Genome of Drosophila melanogaster. Academic Press, Inc., New York

[bib16] Mason J. M. Valencia R. Woodruff R. C. Zimmering S., 1985 Genetic drift and seasonal variation in spontaneous mutation frequencies in *Drosophila*. Environ. Mutagen. 7: 663–676 3930236 10.1002/em.2860070506

[bib17] Miyata T. Hayashida H. Kuma K. Mitsuyasa K. Yasunaga T., 1987 Male-driven molecular evolution: a model and nucleotide sequence analysis. Cold Spring Harb. Symp. Quant. Biol. 52: 863–867 3454295 10.1101/sqb.1987.052.01.094

[bib18] Muller H. J., 1928 The measurement of gene mutation rate in *Drosophila*, its high variability, and its dependence upon temperature. Genetics 13: 279–357 17246553 10.1093/genetics/13.4.279 PMC1200984

[bib19] Muller H. J. Oster I. I., 1963 Some mutational techniques in *Drosophila*, pp. 249–278 in Methodology in Basic Genetics, edited by Burdette W. J. Holden-Day, Inc., San Francisco, CA

[bib20] Rathke C. Baarends W. R. Awe S. Renkawitz-Pohl R., 2014 Chromatin dynamics during spermiogenesis. Biochim. Biophys. Acta 1839: 155–168 24091090 10.1016/j.bbagrm.2013.08.004

[bib21] Shimmin L. Chang B. H.-J. Li W. H., 1993 Male-driven evolution of DNA sequences. Nature 362: 745–747 8469284 10.1038/362745a0

[bib22] Sonnenblick B. P., 1965 The early embryology of *Drosophila melanogaster*, pp. 62–167 in Biology of Drosophila, edited by Demerec M. Hafner Publishing Company, New York

[bib23] Thompson J. N. Jr. Woodruff R. C., 1980 Increased mutation in crosses between geographically separated strains of *Drosophila melanogaster*. Proc. Natl. Acad. Sci. USA 77: 1059–1062 6767240 10.1073/pnas.77.2.1059 PMC348423

[bib24] Thompson V., 1977 Recombination and response to selection in Drosophila melanogaster. Genetics 85: 125–140 402302 10.1093/genetics/85.1.125 PMC1213612

[bib25] Vogel F. Motulsky A. G., 1997 Human Genetics: Problems and Approaches. Ed. 3 Springer-Verlag, New York

[bib26] Wallenfang M. R. Nyak R. DiNardo S., 2006 Dynamics of the male germline stem cell population during aging of *Drosophila melanogaster*. Aging Cell 5: 297–304 16800845 10.1111/j.1474-9726.2006.00221.x

[bib27] Woodruff R. C. Thompson J. N. J., 2005 The fundamental theorem of neutral evolution: Rates of substitution and mutation should factor in premeiotic clusters. Genetica 125: 333–339 16247704 10.1007/s10709-005-4982-7

[bib28] Woodruff R. C. Thompson J. J. N. Seeger M. A. Spivey W. E., 1984 Variation in spontaneous mutation and repair in natural population lines of *Drosophila melanogaster*. Heredity 58: 223–234

[bib29] Woodruff R. C. Huai H. Thompson J. N. Jr, 1996 Clusters of identical new mutation in the evolutionary landscape. Genetica 98: 149–160 8976063 10.1007/BF00121363

